# Cholesterol Screening by Marital Status and Sex in the United States

**Published:** 2009-03-15

**Authors:** Jim P. Stimpson, Fernando A. Wilson

**Affiliations:** Department of Social and Behavioral Sciences, University of North Texas Health Science Center; University of North Texas Health Science Center, Fort Worth, Texas

## Abstract

**Introduction:**

Marital status may be a predisposing factor related to preventive health screenings, which may in part explain the "healthy marriage" effect. This study investigates differences in the likelihood of being screened for cholesterol by marital status for men and women.

**Methods:**

Medical Expenditure Panel Surveys from 2003 through 2005 were used to calculate the likelihood of self-reported cholesterol screening in the past year by marital status and sex. Several rounds of interviews during a 2-year period resulted in a sample of 36,594 US adults.

**Results:**

Most married, widowed, and divorced/separated people reported cholesterol screening in the past year. The highest percentages of people being screened for cholesterol were widowed men (75%) and women (81%). By contrast, 26% of single men and 38% of single women reported cholesterol screening. In multivariate models, being unmarried was associated with lower odds of cholesterol screening among men and women. The lowest likelihood of screening was associated with widowed status for both men (odds ratio, 0.56) and women (odds ratio, 0.53).

**Conclusion:**

Marital status is a predisposing factor for cholesterol screening. Public health interventions aimed at improving preventive screening should focus on social networks, especially family members.

## Introduction

Marriage is associated with positive health outcomes, the mechanisms for which are unclear (1,2). This "healthy marriage" effect results when marital partners are motivated to maintain their health because they feel obligated to other family members who depend on them for economic security and social support (3,4). In contrast, differences in health that are associated with marital status may be viewed as a self-selection effect. People who marry may be healthier than those who remain single, and people who recently were divorced or widowed will initially be less healthy compared with people who are continuously married.

A difference in health-promoting behaviors could partly explain the healthy marriage effect. Married people tend to eat healthier, smoke less, and drink less than unmarried people (5-13). Health-promoting behaviors are likely to be conditional on both sex and marital status. Differences in mortality and morbidity rates are greatest for married men compared with unmarried men, and married women do not have better health compared with women of other marital statuses (14,15).  Limited evidence suggests that women might have some influence over men's screening behavior (16-19). A recent study documented that husbands are screened at approximately the same rate as their wives (13). However, to our knowledge no study has specifically examined whether preventive health screenings vary by marital status and sex.

This study uses nationally representative data to determine whether the likelihood of having cholesterol screening differs by marital status for men and women. We expected women to be more likely to seek cholesterol screening than men. Married men and women were expected to be more likely to seek cholesterol screenings than were unmarried men and women.

## Methods

### Data

We used the most recent data available from the Medical Expenditure Panel Survey (MEPS), a set of large-scale, rotating panel surveys of people, their medical providers, and employers across the United States (20). The panel design of the survey, which features several rounds of interviewing covering 2 full calendar years, enables us to examine changes in health service use, health conditions, and behavior. MEPS has 2 major components: the household component is a nationally representative subsample of people drawn from the National Health Interview Survey (NHIS), and the medical provider component collects information from respondents' health care providers to supplement and verify information provided by respondents in the household component. The breadth of information collected on health service use and predictors of service use places MEPS among the best data sources for understanding differences in prevention screening.

Person-level weights for MEPS adjust for nonresponse over time and reflect Current Population Survey estimates on the basis of 6 variables (race/ethnicity, sex, age, poverty status, region of residence, and urban or rural residence). Because of this weighting scheme, estimates from MEPS are generalizable to the US civilian, noninstitutionalized population. MEPS estimates do not cover households that were created after the NHIS interviews for the respective panels or people not covered by a given MEPS panel, such as people leaving the military, US citizens returning from residence in another country, and people leaving institutions. However, people or households not covered make up a small proportion of the MEPS target population. The final sample size is 36,594 adults with complete information on marital status and cholesterol screening from 2003 through 2005.

### Variables

The outcome variable for this study is cholesterol screening in the past year, measured as a binary self-reported indicator. The independent variable of interest is marital status, which is enumerated separately by people reporting whether they were married, divorced/separated, widowed, or never married. Socioeconomic factors included age, race/ethnicity, family size, urban versus rural residence, region of the country, years of education, occupation, and mean annual household income. Health behavior indicator variables were current smoking status, obesity status, and physical activity (specifically, whether the respondent engaged in moderate to vigorous physical activity at least 3 times weekly). Variables that measured access to health care were type of health insurance and having a usual health care provider. Health conditions were assessed by self-reports of 1) the mean annual number of prescriptions and 2) whether a doctor had ever told the respondent that he or she had had a stroke, heart attack, coronary heart disease, angina, other heart disease, diabetes, or high blood pressure.

### Statistical analysis

Stata version 9 SE (StataCorp LP, College Station, Texas) was used to adjust for the sample weights, strata, and population sampling units. Mean values with confidence intervals for all study variables were calculated by marital status and sex. Multivariate logit regression modeled the odds of having cholesterol screening by marital status for men and women. Correlation ratios were calculated for a stepped-in regression to estimate which group of predictors was most closely associated with screening. Predicted probabilities of screening across age were computed for married and unmarried men and women. The probabilities were generated from the multivariate logit estimates. Interaction terms for age, marital status, and sex were created to test for significant differences in cholesterol screening for married and unmarried men and women by age.

## Results

Most married, widowed, and divorced/separated people reported cholesterol screening in the past year. The highest percentage of people who reported being screened for cholesterol was widowed men (75%) and women (81%). By contrast, 26% of single men and 38% of single women reported cholesterol screening (Table 1 and Table 2).

Unmarried men and women had significantly lower odds of getting screened for cholesterol (Table 3). The lowest likelihood of screening was associated with widowed status for both men (OR, 0.56) and women (OR, 0.53). However, among men the confidence intervals overlapped, which suggests that cholesterol screening does not vary within categories of unmarried respondents. Among women, the confidence intervals for widowed status overlapped for divorced/separated but did not overlap for never married status, which suggests some order in the odds within marital status categories.

Table 4 shows the change in correlation ratios as various categories of screening predictors are stepped into the regression equation. The baseline model shows the correlation ratio for marital status and cholesterol screening. The change in correlation ratios is used to interpret how much the correlation ratio increased from baseline. Therefore, marital status was the strongest predictor of screening for women (0.17) and the second strongest factor for men (0.15). The first adjustment factor stepped in was age. The change in correlation ratios column suggests that, for men, age was the strongest predictor of cholesterol screening (0.22) and, for women, it was the second strongest predictor (0.16). For both men and women, access to care was the next predictor most strongly correlated with screening, followed by health conditions, socioeconomic factors, and health behaviors.

Screening was lowest in younger age groups and increased with age (Figure). Most people aged 35 years or younger reported not having had cholesterol screening. By contrast, most people aged 50 years or older reported cholesterol screening. The difference in screening through age 50 was significant within marital status. Until age 50, unmarried women and men were screened at lower rates. After age 50, no significant difference in screening was seen for men or women regardless of marital status (data not shown).

**Figure.**
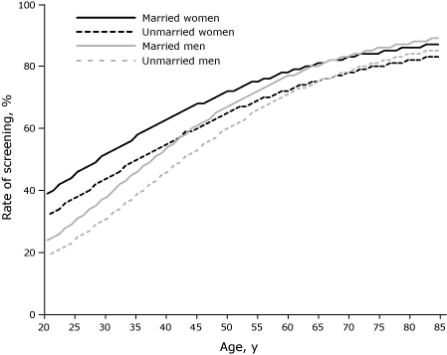
Rate of cholesterol screening during the previous year, by age among married and unmarried men and women, 2003-2005 Medical Expenditure Panel Survey.

**Table:** 

## Discussion

Results from this study indicate that cholesterol screening varies by marital status among men and women. In part, this finding supports the theory that married relationships may lead people to seek preventive care because they are motivated to stay healthy for the sake of their family (3,4). Other research has speculated that a mechanism for the health-promoting effect of marriage is that women's role in the family may be to encourage family members, especially husbands, to be screened (14,15,18). Even if men are not the health gatekeeper for the family, most married men still acknowledge an economic and social obligation to their family, which may motivate them to be screened.

There was a difference in the probabilities of screening across marital status categories before and after adjustment for factors related to screening. Unadjusted results suggested that people in the widowed category had the highest probability of screening, but after adjustment for relevant factors, widowed status was associated with the lowest likelihood of screening. The unadjusted rates for widowed status reflect that older people are more likely to be screened for cholesterol than are younger people. Older people are more likely to need and seek health care than are younger people. In fact, sex differences in cholesterol screening early in life may be explained by recommendations that women get annual gynecologic examinations (at which other kinds of screening may occur), but no annual examination is recommended for young men. Therefore, adjusting for age and other factors associated with screening allowed respondents to be considered equally on the basis of those factors, which suggests that widowed people of the same age, socioeconomic status, health behaviors, and conditions are less likely than married, divorced/separated, and never married people to be screened for cholesterol.

We also studied the strength of the association for the groups of factors associated with cholesterol screening. These results showed that marital status is one of the strongest factors in explaining screening differences, even beyond health behaviors and health conditions. Although marital status had the highest correlation ratio for women and second highest for men, these results should be interpreted cautiously. Marital status is correlated with other factors such as age, income, and health behaviors and conditions. Therefore, the initial correlation of marital status with screening also reflects unobserved correlation with these other factors. However, a reasonable conclusion to draw for this analysis is that marital status predicts the likelihood of cholesterol screening.

We could not adequately test the self-selection hypothesis, which suggests that differences in health by marital status arise from unobservable, idiosyncratic characteristics of people choosing a given marital status. A reasonable strategy to test this hypothesis would be to use prospective panel data over a sufficiently long time period that could capture changes in health behaviors associated with transitions between marital statuses. MEPS is a 2-year, rotating panel data set, and too few cases of marital transitions occurred during a 2-year period to draw reasonable conclusions about the self-selection hypothesis. Therefore, these results should be viewed cautiously, given that the married population may comprise people who are more likely to seek preventive care.

One suggestion to improve screening may be to invite both spouses for the health service visit, which has been associated with increased screening behavior (17). This initiative could be especially helpful for reducing the gap between younger married men and women. Increasing screening among unmarried men may be challenging. Men who are not tied to family responsibilities may feel less obligated to prevent disease compared with men who have family obligations. However, marital status is only 1 component of a person's social network. Recent studies of social networks and health behaviors found that even distant members of a social network can have profound effects on health behaviors (21,22). Therefore, public health interventions should focus on increasing screening among social networks that include children, family members, and friends.

## Figures and Tables

**Table 1:** Characteristics of Men With Complete Information on Cholesterol Screening During the Previous Year and Marital Status, 2003-2005 Medical Expenditure Panel Surveys

**Variable**	Mean or % (95% CI)^a^			

	Married (n = 9,984)	Widowed (n = 445)	Divorced/ Separated (n = 1,749)	Never Married (n = 4,121)
**Cholesterol screening, %**	59.8 (58.0-61.6)	75.1 (70.6-79.6)	51.9 (49.5-54.3)	26.2 (22.9-29.5)
**Age, mean, y**	50.1 (49.7-50.5)	71.4 (69.8-73.0)	49.3 (48.5-50.1)	30.7 (30.5-30.9)
**Race/ethnicity, %**				
Hispanic	11.9 (10.9-12.9)	10.5 (8.3-12.7)	11.6 (6.5-16.7)	15.4 (13.4-17.4)
Black	6.8 (5.2-8.4)	10.0 (9.8-10.2)	13.6 (10.3-16.9)	15.0 (13.0-17.0)
Asian	4.7 (4.1-5.3)	2.7 (1.7-3.7)	1.0 (0.4-1.6)	4.3 (3.1-5.5)
**Family size, mean no.**	3.1 (3.1-3.1)	1.6 (1.4-1.8)	1.7 (1.7-1.7)	2.4 (2.2-2.6)
**Education, %**				
Less than high school	15.3 (13.3-17.3)	33.3 (30.8-35.8)	20.5 (16.6-24.4)	22.7 (21.7-23.7)
High school graduate	30.4 (28.6-32.2)	33.9 (29.0-38.8)	35.1 (31.4-38.8)	31.8 (31.4-32.2)
Some college	21.8 (20.0-23.6)	16.3 (13.0-19.6)	22.2 (15.3-29.1)	24.5 (20.4-28.6)
College graduate	32.2 (30.2-34.2)	16.5 (15.7-17.3)	22.1(19.6-24.6)	21.0 (16.5-25.5)
**Occupation, %**				
Professional	29.4 (28.2-30.6)	5.3 (3.9-6.7)	19.8 (18.0-21.6)	20.9 (17.4-24.4)
Service	18.2 (17.6-18.8)	7.8 (3.5-12.1)	19.9 (17.2-22.6)	33.9 (32.7-35.1)
Not working	23.1 (21.3-24.9)	76.8 (70.5-83.1)	26.5 (25.1-27.9)	27.6 (26.4-28.8)
**Annual income, mean, $1,000s**	42.1 (41.1-43.1)	28.9 (25.0-32.8)	38.7 (37.1-40.3)	23.9 (21.9-25.9)
**Urban area of residence, %**	80.9 (77.2-84.6)	77.8 (70.9-84.7)	79.9 (74.4-85.4)	85.3 (80.4-90.2)
**Region of residence, %**				
Northeast	17.9 (15.9-19.9)	17.7 (9.7-25.7)	16.2 (11.7-20.7)	19.9 (17.4-22.4)
Midwest	22.5 (20.1-24.9)	19.8 (13.9-25.7)	24.2 (19.9-28.5)	21.9 (15.8-28.0)
South	37.0 (35.4-38.6)	39.4 (29.8-49.0)	37.5 (35.5-39.5)	33.6 (27.9-39.3)
West	22.7 (20.7-24.7)	23.1(16.0-30.2)	22.1 (15.8-28.4)	24.6 (22.2-27.0)
**Health behaviors, %**				
Obese	27.7 (25.9-29.5)	21.2 (15.3-27.1)	23.9 (22.3-25.5)	20.3 (18.1-22.5)
Currently smoke	19.0 (18.4-19.6)	25.6 (23.8-27.4)	37.8 (37.0-38.6)	29.3 (27.5-31.1)
Physically active^b^	58.7 (58.1-59.3)	51.8 (48.9-54.7)	63.1 (60.9-65.3)	66.4 (63.1-69.7)
**Access to care, %**				
Private insurance	81.7 (81.3-82.1)	58.0 (50.9-65.1)	60.7 (55.0-66.4)	61.9 (59.5-64.3)
Public insurance	8.7 (8.5-8.9)	33.6 (27.7-39.5)	17.5 (0-60.2)	9.2 (8.4-10.0)
No insurance	9.6 (9.0-10.2)	8.4 (0.4-16.4)	21.8 (20.2-23.4)	28.9 (25.6-32.2)
Have usual provider	80.2 (79.2-81.2)	86.3 (81.6-91)	70.3 (68.7-71.9)	55.7 (54.5-56.9)
**Health conditions**				
Prescriptions, mean annual no.^c^	11.8 (11.4-12.2)	28.4 (26.8-30.0)	11.7 (10.9-12.5)	4.7 (4.5-4.9)
Stroke^d^, %	3.0 (2.8-3.2)	12.5 (9.4-15.6)	3.6 (3.0-4.2)	0.2 (0.2-0.2)
Heart attack^d^, %	4.8 (4.6-5.0)	16.5 (15.5-17.5)	6.5 (6.1-6.9)	0.8 (0.6-1.0)
Coronary disease^d^, %	5.6 (4.8-6.4)	14.7 (14.3-15.1)	5.5 (3.7-7.3)	0.8 (0.6-1.0)
Angina^d^, %	3.3 (2.5-4.1)	7.0 (4.5- 9.5)	4.0 (3.6-4.4)	0.6 (0.4-0.8)
Other heart disease^d^, %	6.4 (5.6-7.2)	17.4 (12.1-22.7)	7.2 (6.6-7.8)	2.4 (2.4-2.4)
Diabetes^d^, %	9.2 (8.4-10.0)	14.3 (12.5-16.1)	8.5 (6.7-10.3)	2.0 (1.8-2.2)
High blood pressure^d^, %	30.3 (29.0-30.9)	57.5 (53.8-61.2)	29.6 (28.4-30.8)	11.7 (10.5-12.9)

Abbreviation: CI, confidence interval.

a Adjusted for sample weights, strata, and population sampling units.

b Defined as engaging in moderate to vigorous physical activity at least 3 times weekly.

c Includes initial purchases and refills.

d Ever told by a doctor that respondent had this condition.

**Table 2:** Characteristics of Women With Complete Information on Cholesterol Screening During the Previous Year and Marital Status, 2003-2005 Medical Expenditure Panel Surveys

**Variable**	Mean or % (95% CI)^a^			

	Married (n = 10,364)	Widowed (n = 2,198)	Divorced/ Separated (n = 3,389)	Never Married (n = 4,344)
**Cholesterol screening, %**	60.2 (59.0-61.4)	80.5 (79.1-81.9)	61.1 (59.1-63.1)	38.4 (35.7-41.1)
**Age, mean, y**	47.0 (46.6-47.4)	72.2 (71.4-73.0)	49.6 (48.8-50.4)	31.1 (30.1-32.1)
**Race/ethnicity, %**				
Hispanic	11.8 (10.2-13.4)	6.3 (5.5-7.1)	11.5 (9.0-14.0)	13.2 (12.2-14.2)
Black	6.8 (5.8-7.8)	11.3 (8.0-14.6)	15.6 (13.2-18.0)	22.3 (19.4-25.2)
Asian	5.2 (4.6-5.8)	2.8 (1.0-4.6)	3.1 (2.5-3.7)	3.7 (2.3-5.1)
**Family size, mean no.**	3.2 (3.2-3.2)	1.6 (1.6-1.6)	2.2 (2.2-2.2)	2.6 (2.6-2.6)
**Education, %**				
Less than high school	13.5 (11.1-15.9)	31.1 (28.7-33.5)	17.5 (16.7-18.3)	21.4 (19.0-23.8)
High school	33.3 (32.7-33.9)	35.8 (32.1-39.5)	35.6 (32.7-38.5)	28.1 (26.3-29.9)
Some college	23.4 (22.4-24.4)	19.3 (18.1-20.5)	25.4 (24.0-26.8)	27.8 (22.1-33.5)
College graduate	29.9 (28.1-31.7)	13.8 (11.8-15.8)	21.4 (19.0-23.8)	22.7(20.2-25.2)
**Occupation, %**				
Professional	29.3 (28.9-29.7)	7.1 (6.9-7.3)	25.4 (22.7-28.1)	24.1 (22.3-25.9)
Service	30.9 (30.5-31.3)	14.2 (13.4-15.0)	39.8 (38.0-41.6)	49.5 (48.5-50.5)
Not working	37.2 (35.8-38.6)	77.5 (77.1-77.9)	30.6 (27.7-33.5)	28.0 (26.8-29.2)
**Annual income, mean, $1,000s**	29.6 (29.0-30.2)	23.0 (22.4-23.6)	29.8 (28.8-30.8)	21.5 (20.7-22.3)
**Urban area of residence, %**	80.5 (76.8-84.2)	78.9 (74.0-83.8)	84.1 (80.0-88.2)	86.2 (82.1-90.3)
**Region of residence, %**				
Northeast	17.7 (16.1-19.3)	23.0 (20.8-25.2)	15.8 (15.2-16.4)	21.1 (19.7-22.5)
Midwest	23.6 (21.6-25.6)	21.6 (19.1-24.1)	22.5 (21.7-23.3)	22.5 (20.1-24.9)
South	36.5 (35.5-37.5)	36.1 (30.0-42.2)	38.1 (35.7-40.5)	34.3 (32.9-35.7)
West	22.3 (20.7-23.9)	19.3 (16.6-22.0)	23.6 (21.6-25.6)	22.0 (20.4-23.6)
**Health behaviors, %**				
Obese	24.2 (23.8-24.6)	27.6 (24.1-31.1)	32.3 (26.8-37.8)	23.8 (23.2-24.4)
Currently smoke	15.4 (14.8-16.0)	13.4 (12.6-14.2)	29.6 (28.6-30.6)	20.7 (19.1-22.3)
Physically active^b^	54.9 (54.7-55.1)	45.5 (44.3-46.7)	53.1 (52.3-53.9)	58.0 (56.6-59.4)
**Access to care, %**				
Private insurance	81.2 (80.2-82.2)	54.6 (49.3-59.9)	63.0 (61.2-64.8)	65.6 (62.5-68.7)
Public insurance	9.3 (8.7-9.9)	40.9 (37.8-44.0)	23.4 (21.2-25.6)	18.0 (14.5-21.5)
No insurance	9.4 (8.2-10.6)	4.5 (2.3-6.7)	13.6 (12.6-14.6)	16.4 (15.4-17.4)
Have usual provider	85.8 (85.2-86.4)	91.5 (89.5-93.5)	84.1 (82.7-85.5)	73.7 (73.1-74.3)
**Health conditions**				
Prescriptions, mean annual no.^c^	13.6 (13.0-14.2)	31.0 (30.2-31.8)	19.1 (17.5-20.7)	8.6 (7.2-10.0)
Stroke^d^, %	1.9 (1.9-1.9)	8.9 (6.9-10.9)	3.9 (3.7-4.1)	0.5 (0.3-0.7)
Heart attack^d^, %	1.4 (1.2-1.6)	8.6 (6.6-10.6)	3.1 (2.5-3.7)	0.6 (0.1-1.2)
Coronary disease^d^, %	2.0 (1.6-2.4)	9.6 (8.2-11.0)	2.7 (1.9-3.5)	0.4 (0.2-0.6)
Angina^d^, %	1.6 (1.2-2.0)	8.2 (7.4-9.0)	2.2 (1.4-3.0)	0.3 (0.3-0.3)
Other heart disease^d^, %	6.3 (5.9-6.7)	16.9 (12.4-21.4)	8.0 (5.5-10.5)	4.5 (4.1-4.9)
Diabetes^d^, %	6.1 (5.9-6.3)	18.1 (17.3-18.9)	9.8 (8.8-10.8)	3.4 (2.4-4.4)
High blood pressure^d^, %	24.3 (23.9-24.7)	62.4 (58.9-65.9)	31.7 (30.1-33.3)	10.7 (9.1-12.3)

Abbreviation: CI, confidence interval.

a Adjusted for sample weights, strata, and population sampling units.

b Defined as engaging in moderate to vigorous physical activity at least 3 times weekly.

c Includes initial purchases and refills.

d Ever told by a doctor that respondent had this condition.

**Table 3:** Multivariate Adjusted^a^ Odds Ratios of Cholesterol Screening During the Previous Year,  by Sex and Marital Status, 2003-2005 Medical Expenditure Panel Surveys

**Marital Status**	Men OR (95% CI)	Women OR (95% CI)
Married	1 [Reference]	1 [Reference]
Widowed	0.56 (0.34-0.77)	0.53 (0.42-0.64)
Divorced/separated	0.76 (0.62-0.91)	0.71 (0.61-0.82)
Never married	0.68 (0.58-0.79)	0.84 (0.72-0.95)

Abbreviations: OR, odds ratio; CI, confidence interval.

a Adjusted for sample weights, strata, population sampling units, year of interview, socioeconomic status, health behaviors, access to care, and health conditions.

**Table 4:** Change in Correlation Ratios^a^ for Multivariate Analysis^b^ of Likelihood of Having Had Cholesterol Screening During the Previous Year, 2003-2005 Medical Expenditure Panel Surveys

**Variable**	Men		Women	

	η^2^	Change	η^2^	Change
Marital status	0.15	NA	0.18	NA
+ Age	0.37	0.22	0.33	0.16
+ Socioeconomic status	0.38	0.01	0.34	0.01
+ Health behaviors	0.38	<0.01	0.35	<0.01
+ Access to care	0.42	0.03	0.36	0.02
+ Health conditions	0.43	0.02	0.38	0.01

Abbreviation: NA, not applicable.

a η^2^ = correlation ratio.

b Adjusted for sample weights, strata, population sampling units, and year of interview.

## References

[1] Kiecolt-Glaser JK, Newton TL (2001). Marriage and health: his and hers. Psychol Bull.

[2] Williams K, Umberson D (2004). Marital status, marital transitions, and health: a sexed life course perspective. J Health Soc Behav.

[3] Umberson D (1992). Gender, marital status, and the social control of health behavior. Soc Sci Med.

[4] Lewis MA, McBride CM, Pollak KI, Puleo E, Butterfield RM, Emmons KM (2006). Understanding health behavior change among couples: an interdependence and communal coping approach. Soc Sci Med.

[5] Broman C (1993). Social relationships and health-related behavior. J Behav Med.

[6] Eng PM, Kawachi I, Fitzmaurice G, Rimm EB (2005). Effects of marital transitions on changes in dietary and other health behaviours in US male health professionals. J Epidemiol Community Health.

[7] Schone BS, Weinick RM (1998). Health-related behaviors and the benefits of marriage for elderly persons. Gerontologist.

[8] Sobal J, Rauschenbach BS, Frongillo EA (2003). Marital status changes and body weight changes: a US longitudinal analysis. Soc Sci Med.

[9] Stimpson JP, Lackan NA (2007). Serum carotenoid levels vary by marital status. J Am Diet Assoc.

[10] Homish GG, Leonard KE (2005). Spousal influence on smoking behaviors in a US community sample of newly married couples. Soc Sci Med.

[11] Meyler D, Stimpson JP, Peek MK (2007). Health concordance within couples: a systematic review. Soc Sci Med.

[12] Stimpson JP, Masel MC, Rudkin L, Peek MK (2006). Shared health behaviors among older Mexican American spouses. Am J Health Behav.

[13] Falba TA, Sindelar JL (2008). Spousal concordance in health behavior change. Health Serv Res.

[14] Chipperfield JG, Havens B (2001). Gender differences in the relationship between marital status transitions and life satisfaction in later life. J Gerontol B Psychol Sci Soc Sci.

[15] Stimpson JP, Kuo YF, Ray LA, Raji MA, Peek MK (2007). Risk of mortality related to widowhood in older Mexican Americans. Ann Epidemiol.

[16] Tudiver F, Talbot Y (1999). Why don't men seek help? Family physicians' perspectives on help-seeking behavior in men. J Fam Pract.

[17] van Jaarsveld, Miles A, Edwards R, Wardle J (2006). Marriage and cancer prevention: does marital status and inviting both spouses together influence colorectal cancer screening participation?. J Med Screen.

[18] Norcross WA, Ramirez C, Palinkas LA (1996). The influence of women on the health care seeking behavior of men. J Fam Pract.

[19] Seymour-Smith S, Wetherell M, Phoenix A (2002). 'My wife ordered me to come': a discursive analysis of doctors' and nurses' accounts of men's use of general practitioners. J Health Psychol.

[20] Cohen JW, Monheit AC, Beauregard KM (1996). The Medical Expenditure Panel Survey: a national health information resource. Inquiry.

[21] Christakis NA, Fowler JH (2007). The spread of obesity in a large social network over 32 years. N Engl J Med.

[22] Christakis NA, Fowler JH (2008). The collective dynamics of smoking in a large social network. N Engl J Med.

